# The Characterization of Dry Fermented Sausages under the “Chorizo Zamorano” Quality Label: The Application of an Alternative Statistical Approach

**DOI:** 10.3390/foods12030483

**Published:** 2023-01-20

**Authors:** Javier Plaza, Carmelo Ávila-Zarza, Ana María Vivar-Quintana, Isabel Revilla

**Affiliations:** 1 Faculty of Environmental and Agricultural Sciences, Universidad de Salamanca, Avenida Filiberto Villalobos 119, 37007 Salamanca, Spain; 2 Department of Statistics, Universidad de Salamanca, Calle Alfonso X El Sabio, 37007 Salamanca, Spain; 3 Food Technology Group, Higher Polytechnic School of Zamora, Universidad de Salamanca, Avenida Requejo 33, 49022 Zamora, Spain

**Keywords:** categorical principal components analysis, physicochemical parameters, sensory parameters, meat product quality, quality brands

## Abstract

The characterization of quality brand meat products, such as “Chorizo Zamorano” dry fermented sausages, involves a wide range of data which makes it necessary to use alternative statistical methodologies. In this study, the feasibility of the Categorical Principal Components Analysis as a multivariate non-linear technique for the characterization of “Chorizo Zamorano” was assessed. The data analyzed were those of eight commercial brands covered by the quality mark over an eight-year period (2013–2020) and included parameters of the physicochemical composition and organoleptic properties of the product. The results showed that “Chorizo Zamorano” has an average moisture content (28.28%), high protein (38.38%) and fat (51.05%) contents, and a very low carbohydrate concentration (1.52%). Results showed that the fat and protein content and the sensory parameters related to external and internal odor appeared to be the studied variables with the greatest influence on the classification of the products according to their quality.

## 1. Introduction

The European Union (EU) quality labels for agricultural products and foodstuffs were introduced in 2013 [1] as a part of EU food quality policy to encourage specific agricultural production, to protect product names from misuse and imitation [2], and to support consumer decisions [3]. From a consumer perspective, EU quality labels increase confidence in food purchases by linking experience qualities, such as taste and convenience, with credence qualities, such as origin or production methods [4]. In addition to the EU quality labels, there are other quality labels in Spain that constitute a regional regulatory framework for agri-food products of differentiated quality. In order to register them at the Spanish Patent and Trademark Office, a favorable report on the Regulation of Use must be issued by an official regional body. This regulation is completed with the Certificate of Product Conformity, which is issued by a certification body responsible for inspecting and verifying compliance with the provisions of the Regulation of Use of the Quality Label in accordance with European Standard EN/45011.

Chorizo Zamorano is one of the meat products which has been awarded the Quality Label denomination. It is a dry fermented sausage from northwest Spain. This product shows significant differences from other products from neighboring areas [5] due, on the one hand, to the careful selection of the raw materials using parts of the carcass which stand out owing to their suitability for pork products, and on the other, its high paprika content with a high proportion of spicy paprika, which makes it highly resistant to oxidation [6]. Its composition, sensory properties, and production characteristics are defined in the “Regulations on the use of the Chorizo Zamorano Quality Label”. The results obtained from the analyses are subjected to an external audit to establish the degree of conformity with the specifications set out in the regulations and to guarantee a certain degree of product homogeneity.

This typicity and homogeneity must be observed not only between producers but also during the year and between years. It is, thus, possible to differentiate Protected Designation of Origin (PDO) or Quality Labelled products from similar ones, although minor differences can be observed between breeds [7], producers [8,9], specific geographical areas [8], and seasons [10,11]. In fact, the differentiation between products of quality brands or other brands [12] or those belonging to different PDOs or Protected Geographical Indications (PGIs) [13] has been extensively researched using a wide variety of multivariate chemometric approaches, such as Principal Component Analysis (PCA), Dissimilarity based (D-PLS), or Orthogonal (OPLS-DA) Partial Least Squares discriminant analyses, Support Vector Machine (SVM), or recognition of patterns by Artificial Neural Networks (ANN), among others [14,15,16,17,18]. In general, these studies used quantitative data and rarely included sensory data, which they derived from quantitative scales. However, the tasting sheets used in some quality labels generate data that are not quantitative, as in the case of Serrano ham (a Spanish TSG product) which uses a scorecard with a six-point quality scale, or in the case of P.D.O. Honey from Corsica, whose evaluation is based mainly on qualitative descriptions of appearance, odor, taste, and texture dimensions [19]. In this case, the use of linear multivariate techniques is not appropriate, and other methodologies need to be investigated.

In this scenario of the simultaneous treatment of different types of variables, i.e., quantitative and qualitative variables, nonlinear multivariate analysis techniques emerge as an effective alternative methodology. These techniques were originally developed by Albert Gifi, the pseudonym of a group of brilliant researchers from the Department of Data Theory at Leiden University. They created a general model based on an alternating least squares algorithm with the objective of analyzing multidimensional relationships in two-dimensional space, whether for nominal, ordinal, or continuous variables, and any combination of these [20]. Among these methodologies, the Categorical Principal Component Analysis uses an optimal scaling process that transforms the category labels into numerical values while the variance accounted for between the quantified variables is maximized [21,22].

As far as we know, there is no scientific literature on which of these methodologies have been implemented in the agri-food sector. This fact paves the way for a new line of research which could be very useful in the regulation of alimentary products protected under a quality brand.

Considering the aforementioned arguments, it has been hypothesized that the characterization of meat products protected under quality brands is essential in order to differentiate them from others with lower quality standards; this improves the profitability and competitiveness of their producers. Moreover, given the mixed nature of the data matrices, i.e., those consisting of quantitative and qualitative variables, nonlinear multivariate techniques could have remarkable potential to achieve this characterization. The aim of this study is, therefore, to characterize dry fermented sausages belonging to the “Chorizo Zamorano” Quality Label by the simultaneous treatment of physico-chemical and organoleptic data using a specific nonlinear multivariate technique known as the Categorical Principal Components Analysis.

## 2. Materials and Methods

### 2.1. Samples

In this study, a total of 64 different commercial samples of dry fermented sausage, henceforth chorizo, supplied by the “Association for the promotion of the Chorizo Zamorano” Quality Label were analyzed. These samples were individually and periodically provided at the optimum consumption moment by the eight producers covered by the quality label during eight years from 2013 to 2020 (N = 64 samples; 1 sample/producer/year), according to the design established by the Regulatory Board of the Chorizo Zamorano Quality Label [23]. The samples were produced according to the ingredients and procedures established by the Regulatory Board of the Chorizo Zamorano Quality Label, which establishes that this product is a dry fermented sausage produced from fresh meat of white-layered fatty pork with carcasses over 85 kg and from which the loin, shoulder, ham, belly, lean meat, and streaky bacon are used, together with the addition of sweet or spicy paprika belonging to the “Pimentón de la Vera” Denomination of Origin, salt, garlic, and oregano. The raw materials are chopped up (in pieces of 8 mm to 12 mm), mixed with the remainder of the ingredients, left to stand, and stuffed into natural cow or pig gut casings; the chorizos are then kept in a drying room with a relative humidity of between 70 and 85% at a temperature of between 7 and 12 °C for at least four weeks.

### 2.2. Physicochemical Analysis

All the analyses were performed in the “Estación Tecnológica de la Carne del Instituto Tecnológico Agrario” (Meat Technology Station of the Agricultural Technology Institute) belonging to the Castilla y León Council (Guijuelo, Castilla y León, Spain). When the samples reached the optimum time for sale and consumption, at the discretion of each producer, they were vacuum-packed (Tecnotrip V220) in pouches of polyethylene and kept refrigerated at 4 °C until analysis. Moisture content was determined by drying following the ISO 1442:1997 method [24]. Fat content was evaluated by Sohxlet extraction with diethyl ether according to the ISO method 1443:1973 [25]. Kjeldahl N determination and protein calculation was performed using the AOAC official method 990.03 [26]. Water activity was measured using a Decagon CX-2 AQUALAB hygrometer (Decagon Devices Inc., Pullman, WA, USA) at 20 °C. The pH was measured using a Crison model 507 pH meter with a puncture electrode. Nitrate content was determined by high-performance liquid chromatography (HPLC) (Agilent 1100/HP, Santa Clara, CA, USA) using a method proposed by Merino et al. [27]. Nitrite content was determined according to ISO method 3091:1975 [28]. Total carbohydrates were quantified by the Luff–Schoorl method [29]. The determination of hydroxyproline content was described by Bonnet and Kopp [30]. The chloride content (NaCl) was carried out following the 925.56 AOAC method [31]. All the determinations were carried out in triplicate.

### 2.3. Sensory Analysis

The sensory analysis of the samples was carried out by a panel of ten judges selected in advance with a wide experience in the evaluation of meat products. The selection, training, and monitoring of the ten judges were carried out in accordance with European standard EN ISO 8586:2014 and international standard ISO 8586:2012. Since the work described was carried out over a period of eight years, some of the members have changed; however, any new members were selected and trained in sensory analysis. The samples were individually labeled with three-digit random numbers. All the sessions (eight samples each year, four samples evaluated in each session) were held in a sensory analysis laboratory equipped with individual booths using the sensory card designed by the Regulatory Board of the Chorizo Zamorano Quality Label (Table 1). This card includes the evaluation of external parameters (assessed in whole intact products), the evaluation of the cutting area (assessed in a 10-cm section of the chorizos cut across their axis), and parameters related to the tasting phase (assessed in 1-cm thick slices taken 3 cm from the end of the chorizos) using a five-point scale (1 to 5) according to Table 1.

### 2.4. Statistical Analysis

In this study, two different types of variables were collected: on the one hand, quantitative variables correspond to the physicochemical parameters of chorizo, and on the other hand, qualitative variables are associated with the sensory parameters of the product. The relevant but different information which the two types of variables provided in the study suggested that all of them should be analyzed simultaneously in a multivariate manner as otherwise, there is a significant probability of missing part of that information. For this reason, the commonly used multivariate techniques were soon discarded as most of them were developed for the analysis of either only quantitative or only qualitative data. Instead, we chose to use the not-so-well-known nonlinear multivariate techniques from the Optimal Scaling approach, also known as GIFI techniques [20,32], which allow the modeling of non-linear relationships between variables. In particular, among the GIFI techniques, the Categorical Principal Components Analysis [33], also known as PRINCALS or CATPCA, was considered to be the best fit for the specific characteristics of our data. The consequent reduction of the multivariate problem to an affordable scale makes the observation of the positioning of the product evaluations feasible, together with their relationship with the physicochemical characteristics involved, all of this from a new innovative perspective. Statistical processing of the data was performed using IBM-SPSS Statistics 26 software (IBM, Chicago, IL, USA).

## 3. Results and Discussion

### 3.1. The Physicochemical and Sensory Characteristics of Chorizo Zamorano

The quality marks establish in their regulations the performance of simple analyses, which hinders the identification and discrimination of the different producers that comprise it. Table 2 shows the mean, minimum, maximum values, and mean standard error (MSE) of those parameters. All parameters showed a wide range of variation due to the different brands and years; thus, moisture ranged between 18.83 and 40.20, fat between 40.10 and 60.20, chlorides between 3.00 and 6.80, and water activity between 0.80 and 0.92. However, the mean values of all these parameters were within the range previously reported for different types of chorizo [34,35,36,37]. The protein content varied between 30.40 and 49.10, and the mean value was higher than those reported for other chorizos [34,36,38] with low values of hydroxyproline (0.21–0.66), confirming that producers used good quality meat with low connective tissue content. The low carbohydrate mean value (0.59–4.27) was related to the low or non-existent carbohydrate content included in these products by the manufacturers. The pH showed a wide variation (4.74–6.12), and the mean value was at the upper end of the range reported for chorizo [34,35,36,38], which indicates that Chorizo Zamorano is characterized by a relatively high pH. Nitrate values were below 40 ppm, and nitrite values were below 10 ppm. Both parameters were below the maximum limits set by the regulatory board and showed a low variation between samples. The amounts found in all samples were also lower than those previously reported for the chorizo [38].

Table 2 also shows the mean, maximum, minimum, and standard deviations of sensory parameters. It is important to stress that in contrast to other studies using structured scales for which the higher the perceived sensation of each of the sensory attributes, the higher the score [36,37,39], the “Chorizo Zamorano” Quality Label used a scale in which the highest score (5) corresponded to the ideal intensity of the parameter. This means that for some of the parameters, the highest score corresponded to an average intensity or a low value, such as for chewiness (Table 1), which makes it difficult to compare the sensory characteristics of this product with those of other Spanish chorizos.

As a result, all the values were between 4 and 5, with hardness and chewiness showing the lowest average values (which corresponded to a firm product but with low chewiness), while the highest average values were those of the ease of removing the casing and meat mass binding (which corresponded to a product with a high level of binding between meat and fat without holes with a casing which was easily removed). The intensity of the internal odor and flavor showed high average values (4.27 and 4.19), which meant that these products showed an average intensity for these parameters.

### 3.2. Data Pretreatment

Since the Chorizo Zamorano Quality Label was created, the five-point scale proposed by its Regulatory Board (Table 1) has been used by the experts of the sensory panel to determine whether a certain product meets the necessary requirements for protection under this quality label. However, from a statistical point of view, we found that this data categorization was not entirely appropriate as the extreme categories (1 and 5) had not been selected in any of the cases, which means a large degree of representativeness is lost. This finding is in accordance with the statistical problem known as the central tendency bias [40], which explains why panelists tend to place their responses in the middle categories of a Likert scale. However, this bias could have been eluded by expanding the number of response categories in the sensory scale. Eliminating this response bias is essential in order to directly associate the precision of the registered variability of the data with the accuracy of the sensor analysis [41]. Moreover, as mentioned above, the scales of measurement in the different variables were heterogeneous and, therefore, not at all susceptible to being subjected to multivariate analysis.

In order to address this issue and to make the data amenable to a non-linear multivariate analysis (CATPCA), a recodification of the database was carried out (Table 3) since expanding the number of response categories was not an option after the sensory analysis was conducted. For all the sensory variables studied, a three-class scale was proposed, with class 1 corresponding to the lowest quality and class 3 to the highest quality. Subsequently, the chorizo samples were classified according to the number of variables in each of the types defined in Table 3, considering 9 as an upper and lower threshold, in terms of the number of variables, with a high enough degree of reliability to classify the different chorizo samples.

This new classification could be directly associated with the actual quality of each sample (Table 4), distinguishing between products of moderate, good, very good, or excellent quality.

Once the data were properly recorded, the Categorical Principal Components Analysis was performed using all the variables of the data matrix, i.e., the eight quantitative variables from the physicochemical analysis and the 11 categorical variables from the sensory assessment. At the beginning of the process, the analytical variables were transformed into numerical variables while the sensory variables were transformed into ordinal variables, all with a weighting of 1. Following the criterion of the amount of variability explained, it was decided to maintain the first two dimensions among the resulting ones. The Principal per Variable standardization method was chosen. In order to improve the interpretability of the graphical result, an orthogonal Varimax rotation with Kaiser standardization was selected.

### 3.3. Results of the Categorical Principal Components Analysis

**Figure 1:**
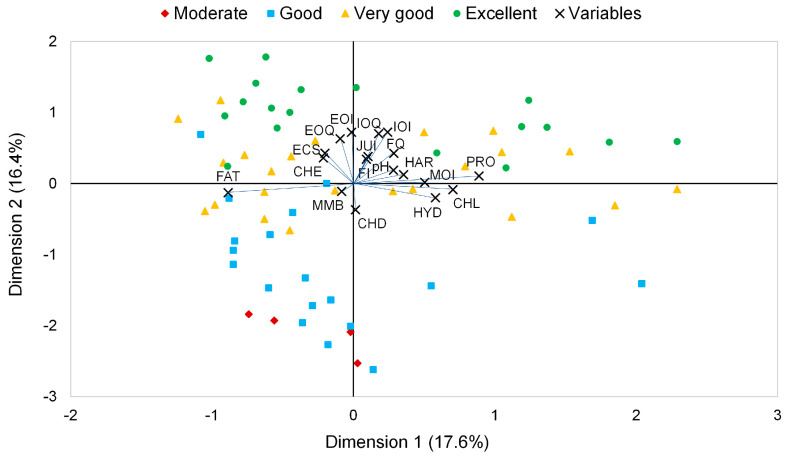
Scatter plot that shows the first plane result, which together covers 34% of the total variance. Variables are shown by lines, where MOI: moisture (g/100 g Dry Matter (DM)), PRO: protein (g/100 g DM), FAT: fat (g/100 g DM), CHL: chlorides (g NaCl/100 g DM), HYD: hydroxyproline (g/100 g DM), CHD: carbohydrates (glucose/100 g DM), EOQ: external odor quality, EOI: external odor intensity, ECS: ease of casing separation, MMB: meat mass binding, IOQ: internal odor quality, IOI: internal odor intensity, HAR: hardness, CHE: chewiness, JUI: juiciness, FQ: flavor quality and FI: flavor intensity.

Figure 1 shows the main graphic result of the Categorical Principal Components Analysis in which the chorizo samples were analyzed, and the variables studied were simultaneously represented in a bi-dimensional space. Dimensional reduction through this optimal scaling technique resulted in a two-dimensional space solution. While the most influential variables in dimension 1 were two physicochemical parameters, i.e., fat and protein, in dimension 2, the position of the samples was essentially determined by a wide range of sensory parameters, mainly by those related to internal and external odor assessment although carbohydrates also had a relevant influence on this dimension. Furthermore, in order to find a grouping trend according to the quality of the samples, the positions of the chorizo samples were grouped based on the four previously mentioned categories (Moderate, Good, Very Good, or Excellent quality products) which were created after recoding the database (Figure 1).

**Figure 2:**
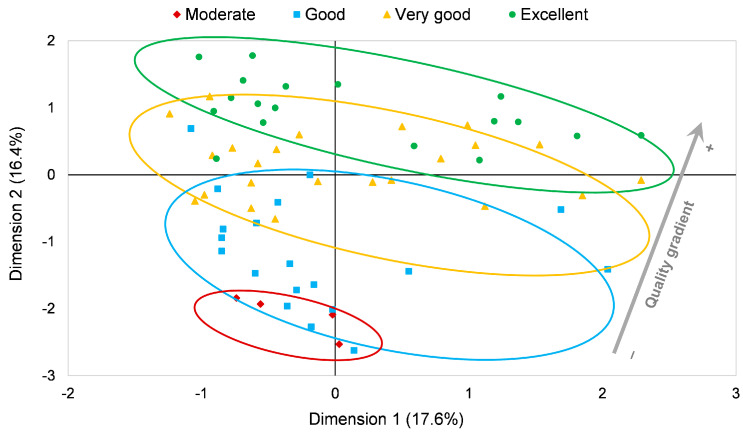
Quality gradient observed in the distribution of the “Chorizo Zamorano” samples in the first bi-dimensional plane.

The initial screening of the results shown in Figure 1 allowed the elucidation of a quality gradient in the distribution of the samples in the bi-dimensional space (Figure 2). Specifically, samples with the lowest quality, named Moderate (red), were located on the lower part of the graph, in contrast to the highest quality samples classed as Excellent (green), which were found on the upper part of the scatter plot.

This result suggests that an odor, both external and internal, of medium intensity and balanced in its predominant notes was what most influenced the excellence of the product, while a high content of carbohydrates and hydroxyproline could be related to lower quality. Indeed, total fat content and chlorides, together with meat mass binding variables, are also associated with negative values of Dimension 2. The presence of hydroxyproline is used as an analytical criterion to assess the amount of collagen from the skin, tendons, cartilage, and ligaments [42] so that high values of this parameter are correlated with the use of second-quality meat [38]. A higher carbohydrate content in the final product may be related to the initial addition of a higher amount of less easily fermentable sugars [38], which leads to lower development of fermentation, producing a lower degradation of carbohydrates and lower quantity and diversity of aromatic compounds [43].

Regarding CHL, sodium chloride has some positive effects on the quality properties of meat products, as it promotes the characteristics of aroma and salty taste development and improves the release of aroma substances from the food matrix, and has a salting-out effect [44]. However, high levels of salt could result in a very salty taste and, in certain cases, could inhibit oxidation, lowering enzyme activity [45] and decreasing the aroma production [43].

On the other hand, previous works have shown that, in general, greater sensory characteristics were observed for low- and medium-fat sausages [46] because higher fat contents reduce hardness, fat–lean cohesiveness, odor intensity, and spice odor [39].

Finally, meat mass binding was correlated with good and very good products, probably because high values of this parameter were not correlated with the desired flavor profile, which is the most important variable for excellent quality products.

This result coincides with those previously obtained by Ambrosiadis et al. [47], who reported that flavor was the main quality index, while fat was considered to be a negative quality index. In fact, fat and protein were sited in opposite parts of Dimension 1 in agreement with previous studies, which found that the higher the fat content, the lower the moisture and protein content [47]. Moreover, according to Figure 1, protein had a higher correlation with excellent samples. The results also showed a positive correlation between protein, moisture, and chlorides, while Ambrosiadis et al. [47] reported that ash was a negative quality index. Previous studies have shown the influence of fat content on sausage quality; a high content produced poor color development [48] and resulted in products that were soft and too fatty [47,49]. This result points out that the CAPTCA analysis, which uses categorical variables which cannot be correlated between them or with quantitative variables, showed results comparable to those of the traditional statistical analysis.

Currently, traditional statistical approaches based on a single food quality attribute (univariate methods) have been almost completely replaced by linear multivariate data techniques, the potential of which, in the study of food quality changes, has been widely proved [50,51]. Furthermore, some non-linear approaches have been taken in food analysis, among which the most widely used are artificial neural networks which are commonly used to predict sensory characteristics [52] or to determine food adulteration [53,54]. However, none of these methodologies have yet been able to overcome the inherent limitation of the mixed nature of the data matrices commonly used in the characterization of agri-food products. In this context, our results have evidenced the capability of the Categorical Principal Components Analysis to extract relevant information from the simultaneous treatment of quantitative and qualitative variables.

Analogous to the quality gradient, a potential gradient generated by samples produced in different years or by different producers was also explored. However, given the homogeneity inherent to products that have been produced under the control of a regulatory board of a quality mark, neither a year nor a producer stands out over the rest in terms of chorizo quality. In other words, there was no clear pattern that identified a constant high- or low-quality year or producer. These results differed from those obtained in previous studies in which significant differences due to these factors were reported [8,55]. On the contrary, our results agreed with other studies, which found that if the manufacturing process was stable, the physicochemical characteristics and the organoleptic qualities were consistent throughout the seasons [56,57].

## 4. Conclusions

The characterization of quality label products is crucial in order to improve the competitiveness of producers and hence, their profits. Chorizo Zamorano has turned out to be a high-protein meat product with a quite homogeneous physicochemical composition between producers over the years. Furthermore, this work has proved the feasibility of a characterization performed using simple analytical parameters. In accordance with the results obtained, fat and protein content, in addition to external and internal odor, were the variables that determined the classification of the chorizo samples according to the quality gradient proposed.

The Categorical Principal Components Analysis has proven to be a suitable tool to use in food products when both qualitative and quantitative data are available. Owing to the way it operates, it is possible to integrate laboratory data corresponding to physicochemical analysis with qualitative variables deriving from the sensory analysis of the products, which offers a global perspective for the characterization of food.

As far as we are concerned, there are no previous works in which sensory and physicochemical parameters were simultaneously analyzed. The results obtained with the CATPCA multivariate technique might be considered really satisfactory. However, further studies must be conducted to gain a deeper insight into the feasibility of using this technique in the agro-food field.

## Figures and Tables

**Table 1:** The sensory card used in the evaluation of dry-fermented sausages of the “Chorizo Zamorano” quality label.

Attribute	Definition	Minimum (1)	Maximum (5)
External evaluation			
External odor quality	The presence of the typical odor in a balanced way, characterized by hints of paprika, gut and mold without any of them predominating	Defective or atypical	Typical
External odor intensity	The intensity of the overall odor of the sample before cutting	Low or very high intensity	Medium intensity
Evaluation of the cutting area			
Ease of casing separation	Degree of easiness displayed when removing the casing of the slice	Difficult	Easy
Meat mass binding	The level at which the granules of fat and meat are bonded and the presence of the holes inside	Low binding big holes	High binding without holes
Internal odor quality	The presence of the balanced and typical odor, slightly acid and characterized by hints of paprika and spices (oregano, garlic)	Not balanced or atypical	Balanced and typical
Internal odor intensity	The intensity of the overall odor of the sample	Low or very high intensity	Medium intensity
Evaluation during consumption			
Hardness	The force necessary to penetrate the meat with the incisors	Tender	Tough
Chewiness	The number of times the sample must be chewed before it can be swallowed	Many	Few
Juiciness	The amount of juice given off by the sample when chewed	Dry	Juicy
Flavor quality	The presence of the balanced and typical flavor in cured meat, slightly acid and characterized by hints of paprika and spices	Not balanced or atypical	Balanced and typical
Flavor intensity	The intensity of the overall flavor of the sample	Low or very high intensity	Medium intensity

**Table 2:** Results of the physicochemical and sensory analysis of dry-fermented sausages of the “Chorizo Zamorano” quality label.

Parameter	Average	Minimum	Maximum	MSE *
Moisture (g/100 g DM)	28.28	18.83	40.20	0.50
Protein (g/100 g DM)	38.43	30.40	49.10	0.51
Fat (g/100 g DM)	51.05	40.10	60.30	0.62
Chlorides (g NaCl/100 g DM)	4.80	3.00	6.80	0.09
Hydroxyproline (g/100 g DM)	0.42	0.21	0.66	0.01
Carbohydrates (g glucose/100 g DM)	1.52	0.59	4.27	0.12
pH	5.34	4.74	6.12	0.04
Water activity	0.86	0.80	0.92	0.00
External odor quality	3.92	3.10	4.80	0.05
External odor intensity	4.01	3.20	4.70	0.05
Ease of casing separation	4.27	1.70	5.00	0.07
Meat mass binding	4.22	2.80	4.90	0.06
Internal odor quality	4.20	3.30	4.80	0.05
Internal odor intensity	4.18	3.30	4.80	0.05
Hardness	3.79	2.80	4.70	0.06
Chewiness	3.79	3.00	4.70	0.05
Juiciness	4.01	3.10	4.70	0.05
Flavor quality	3.98	2.40	4.70	0.06
Flavor intensity	4.16	3.40	4.90	0.04

* MSE: mean standard error.

**Table 3:** Recodification of the sensory card used in the “Chorizo Zamorano” quality label.

Attribute	1 (Lowest Quality)	2	3 (Highest Quality)
External evaluation			
External odor quality	Defective or atypical	Medium	Typical
External odor intensity	Extreme or absent intensity	High or poor intensity	Medium intensity
Evaluation of the cutting area			
Ease of casing separation	Difficult	Medium	Easy
Meat mass binding	Low binding with big holes	Moderate-binding	High binding without holes
Internal odor quality	Not balanced or atypical	Moderately balanced	Balanced and typical
Internal odor intensity	Extreme or absent intensity	High or poor intensity	Medium intensity
Evaluation during consumption			
Hardness	Very tender or tough	Tender or tough	Medium
Chewiness	A lot of	Quite a few	A few
Juiciness	Dry	Moderately juicy	Juicy
Flavor quality	Not balanced or atypical	Moderately balanced	Balanced and typical
Flavor intensity	Extreme or absent intensity	High or poor intensity	Medium intensity

**Table 4:** Quality classification criteria of the “Chorizo Zamorano” samples.

Criterium	Product Quality
Number of variables with a score of 1 ≥ 9	Moderate
Number of variables with a score of 1 > Number of variables with a score of 2 or 3.	Good
Number of variables with a score of 1 < Number of variables with a score of 2 or 3.	Very good
Number of variables with a score of 2 or 3 ≥ 9	Excellent

## Data Availability

The data presented in this study are available on request from the corresponding author.
